# Assessing changes in knowledge, attitudes, and intentions to use family planning after watching documentary and drama health education films: a qualitative study

**DOI:** 10.1186/s12978-022-01370-5

**Published:** 2022-03-12

**Authors:** Vincent Mubangizi, Jane Plastow, Florence Nakaggwa, Haven Nahabwe, Sylvia Natukunda, Fiona Atim, Brenda Mawere, Matthew Laughton, Ingrid Muller, Judith Owokuhaisa, Sabine Coates, Isabella Chambers, Clare Goodhart, Merlin Willcox

**Affiliations:** 1 Mbarara University of Science and Technology, P.O. Box 1410, Mbarara, Uganda; 2 School of English, The University of Leeds, Leeds, UK; 3 Clarke International University, Kampala, Uganda; 4 Bwindi Community Hospital, Kanungu District, Uganda; 5 We are Walukuba Drama Group, Jinja, Uganda; 6 Primary Care Research Centre, School of Primary Care, Population Sciences and Medical Education, University of Southampton, Southampton, UK; 7 University of Bristol Medical School, Bristol, UK; 8 Royal College of General Practitioners, London, UK

**Keywords:** Post-partum, Family planning, Uganda, Implant, Drama, Documentary, Films, Health promotion

## Abstract

**Background:**

There is a paucity of literature on the effectiveness of drama or documentary films in changing knowledge, beliefs, attitudes, and behavior of people towards family planning. This study aimed to compare and assess the acceptability of health promotion films based on documentary or drama, and their effect on knowledge, attitudes, and intention to use family planning.

**Methods:**

We developed short documentary and drama films about contraceptive implants, using the person-based approach. Their acceptability was assessed in focus group discussions with younger women below 23 years, women over 23 years, men of reproductive age, and health workers in four different areas of Uganda (Bwindi/Kanungu, Walukuba/Jinja, Kampala, and Mbarara). Transcripts of the focus group discussions were analyzed using thematic analysis, to generate themes and examine the key issues. We assessed changes in knowledge, attitudes, and intentions to use family planning after watching the films.

**Results:**

Sixteen focus groups with 150 participants were carried out. Participants said that the documentary improved their knowledge and addressed their fears about side effects, myths, and implant insertion. The drama improved their attitudes towards the implant and encouraged them to discuss family planning with their partner. The final versions of the documentary and the drama films were equally liked.

**Conclusions:**

Viewing a short documentary on the contraceptive implant led to positive changes in knowledge, while a short drama improved attitudes and intentions to discuss the implant with their partner. The drama and documentary have complementary features, and most participants wanted to see both.

## Plain Language summary

Thousands of women and children in Uganda die every year due to problems related to pregnancy and childbirth. About 20% of these deaths are avoidable by better use of contraception. Many women are reluctant to begin contraception because of fears and myths about side-effects, and because they need the agreement of their husbands. The aim of this research was to study whether health education films could address these fears, and to compare a short documentary with a short drama film. We produced two health education films (a documentary and a drama) in two local languages with the involvement of local people. Films aimed to (1) dispel some myths on contraception, (2) encourage men to attend at least one antenatal clinic with their wives, and (3) discuss with a health worker whether they would like a method of family planning after the delivery. We showed these films to focus groups of local women, men and health workers in four contrasting areas of Uganda. The people taking part in the focus groups discussed their reactions to the films, whether they had learned anything from them, whether their attitudes towards family planning had changed as a result, and whether they intended to discuss this with their partner. Viewing a short documentary on the contraceptive implant improved knowledge, and short drama films improved attitudes and intentions to discuss the implant with their partner. The documentary and drama had different advantages, and most participants wanted to see both.

## Background

Uganda has a very high maternal mortality ratio (336 maternal deaths per 100,000 live births) and child mortality rate (64 child deaths per 1000 live births) [1]. Reducing these deaths by 2030 is part of goal three of the Sustainable Development Goals [2].

Although the desired fertility rate is 4.3 children per woman, the actual fertility rate of 5.4 children per woman is higher [1]. A third of sexually active women have an unmet need for family planning [3], leading to many unplanned pregnancies, and consequently, unsafe abortions. Up to 26% of maternal deaths are due to unsafe abortion, and many survivors suffer complications [4]. Through education on the usage and availability of contraceptives, the unmet need may be reduced, resulting in fewer pregnancies and less risk of maternal death. The successful use of family planning leads to better child spacing contributing to a reduction in child mortality [5, 6].

The majority of those using contraception in Uganda use the injection, which lasts only 3 months. It is followed in popularity by implants, and then intrauterine contraceptive devices (IUD) [3]. These are non-user-dependent long-acting reversible contraceptives (LARCs) and have greater efficacy than other methods [7]. The first two priorities of the Ugandan government’s family planning implementation plan are to increase uptake of contraception and to address myths, misconceptions, and side effects to prevent unwanted pregnancies [8]. In line with this, we have developed films focusing on LARCs, particularly the implant.

Misconceptions over the side effects of the implant are one of the barriers to its use [9, 10], which can be addressed through education and clarification, as shown in many studies [10–12]. The need for women to discuss family planning with their husbands before use is another major issue [10] in postpartum family planning. With many husbands absent at the time of delivery, women are reluctant to use immediate post-partum family planning, fearing their husbands’ disapproval. Couples also want to hear from current users of contraception about their personal experiences [10].

Contraceptive use decreases as the education level decreases [12]. In Uganda, literacy rates are very variable according to the area. On average, 68% of women and 80% of men can read, but this drops by nearly 10% in rural areas, where contraceptive uptake is at the lowest and unmet need for family planning is highest [1]. Non-written media are therefore needed to target the largest possible audience.

To address key barriers to contraceptive uptake in Uganda, we developed films using the person-based approach [13] as a behaviour change intervention. Using films is advantageous since they can easily be scaled up and used across all regions, in some health-based or social settings and in a variety of standardized layouts and languages, to have a much greater impact than more localized promotions as seen in other studies [14]. The films were designed to be engaging, persuasive, and informative [15]. Films focussing on health behaviour change can be documentaries based on real people or drama using fictional characters [16].

Although both health education documentaries and dramas have been made on family planning, we are not aware of any research comparing the acceptability of these two different styles. People may find it more persuasive to listen to real people’s experiences on family planning, but a drama may be more entertaining and engaging. At present, there appears to be no evidence on the relative effectiveness and acceptability of dramas or documentaries in changing knowledge, beliefs, attitudes, and practice of people towards family planning. Thus, there is a need to explore and investigate whether drama or documentary films have greater appeal to audiences in this setting.

We developed both documentary and drama films to address specific barriers to uptake of the contraceptive implant, which came to light in our previous qualitative study [10]. The films were made in two local languages, Luganda (spoken in central Uganda) and Rukiga/Runyankole (spoken in South-Western Uganda). The objective of this study was to assess changes in knowledge, attitudes, and intentions to use family planning after watching health education films based on real people (documentary) and fictional characters (drama).

## Methods

### Study design

As part of the person-based approach to intervention development, this study was designed to enable optimization of the films. A qualitative study design was used to gain an in-depth understanding of people’s experiences and views of the intervention films. Since the films targeted very personal and socially based issues, in-depth feedback was required to modify them effectively. The in-depth nature of qualitative analysis provides rich views of multiple individuals. Also, verbal feedback overcomes the issue of low literacy rates [1], which would otherwise limit feedback from some individuals from written data collection methods such as surveys [17].

### Development of the films

The development of the films using a person-based approach and modification of the films are described in more detail elsewhere (Willcox et al., Adapting the Person-Based Approach to the development of health education films on family planning in Uganda, a low-income country, submitted) This paper focuses on changes in knowledge, attitudes, and intentions to use family planning after watching the optimised documentary and drama films. In brief, four films were made: a documentary and a drama, each in two different languages (Luganda and Runyankole). Each film was improved based on the feedback from a first round of focus group discussions, and the final films lasted 11–18 min. For both films, the researchers identified concerns/fears to address, encouraging couples to discuss LARCs (especially the implant) and post-partum family planning. All of the films were filmed and edited professionally. The drama revolves around two women who used family planning without discussing it with their husbands. One of these is experiencing side effects, and her mother-in-law brings her to the local woman who recommended the implant. An argument occurs, and a nurse appears and calms the group by providing more information about family planning.

The documentary was unscripted, so the two versions had some minor variations, for example, the Luganda version did not address myths around vaginal dryness or lack of libido, which were mentioned in the Runyankole version. Women who have successfully used an implant including in the immediate post-partum period, and their partners, gave testimonies on the advantages of using family planning, why they got the implant, how it has helped them, experiences using it, and how they dealt with side-effects. Health workers gave information on side-effects and how to manage them, encouraging men to agree on a method of post-partum family planning during the antenatal period. The documentary also shows the implant insertion and removal procedures.

### Study sites

Focus groups were held in four different areas to give a range of viewpoints and feedback in February–March 2019. We held focus groups to discuss the Luganda films in (1) Kisugu Health Centre III, in Makindye division (Kibuli, Namuwongo, and Kabalagala wards), Kampala—serving deprived urban areas within this part of the capital, and (2) Walukuba Health Centre IV, Jinja—serving a deprived suburb of Jinja town. To discuss the Runyankole/Rukiga films, we held focus groups in (3) Mbarara town—an urban area in the southwest of Uganda, and (4) Kayonza, Kanyantorogo and Mpungu sub-counties, Kanungu district—remote rural areas with a sparse population. In each area, focus group discussions were held in a quiet place, such as a meeting room in the hospital or health centres, from which the participants had been recruited.

### Selection and recruitment of participants

Four different focus groups were carried out in each study site. We purposively selected the following participants in each study area: (1) Pregnant or postpartum women attending antenatal or postnatal clinics or baby immunization clinics at health facilities, categorized into younger women (age below 23 years) and women aged 23–49 years. (2) Men of reproductive age attending the health facility or in the community, and (3) Health workers (particularly those in charge of antenatal and postnatal clinics, and of family planning services).

Potential participants from these groups were approached by a member of the study team in waiting areas for antenatal, postnatal, and immunization clinics at health centres. They provided the study information sheets, and the study was explained to them. If they wished to continue, they were recruited and consented to be part of the focus group discussion for their demographic group. We purposively sought a range of women of different ages and parities and aimed to have up to 8–12 participants in each focus group. We held separate focus group discussions with adolescent girls because they represent a particularly vulnerable group, who may also be reluctant to speak openly in the presence of older women. We recruited men of reproductive age who are married or sexually active, who happened to be visiting the health facility for other reasons, or at meeting places in the community.

To find health workers, the researchers contacted a selection of different types of health centre in each study area and asked them whether they would be willing to take part in a focus group discussion, and if so, when would be a convenient time for them. The focus group discussions were thus held at their preferred time so as not to interrupt service delivery. The potential participants were given study information sheets and the study was explained to them. If they wished to continue, they were invited to sign a written consent form. It was difficult to find different health workers who had not participated in first round, so in some cases, the same health workers were invited to more than one round.

### Data collection

Data was collected through focus group discussions using open-ended questions in line with the study objectives. The study aims were explained and participants were reminded that there are no right or wrong answers and that everyone’s views are important. Participants were reminded that the focus group discussion would be recorded. Initially, before watching the films, participants were asked about their current views on family planning and discussing this with their partner. We asked the participants whether they had heard about the contraceptive implant, whether they had any concerns about the implant, and whether they would feel happy to use an implant. The participants were then shown the films, in a random order (sometimes the drama first, sometimes the documentary first). Using an interview guide, participants discussed their views, likes, and dislikes of the films, the preferred film, and why, along with suggestions for improvements for the films as a whole and the messages throughout. Participants also discussed couples’ counselling and communication with partners around family planning. The discussions continued till no new information was being said. On average, focus group discussions lasted 45 min.

### Quality assurance of data collection

Focus group discussions were moderated by trained and experienced qualitative research assistants. The research assistants had no prior relationship with participants. They had also been trained on relevant aspects of good clinical practice, in particular taking consent and respecting confidentiality. Focus groups were carried out in the local language in each area and facilitators were fluent in either Luganda or Runyankole. Some of the health worker focus groups were carried out in English if all participants were fluent.

### Data management and analysis

Preliminary feedback, based on interview notes, was given immediately after the focus groups on the main points raised and used to assist in the re-filming of later versions of the documentary and drama. Additionally, the focus group discussions were recorded on a digital voice recorder and then transcribed directly into English. The transcripts were coded by three researchers (MW, VM, and JO) who met and agreed on a coding frame. The transcripts were analysed using thematic analysis as described by Braun and Clarke [18] to develop and explore codes and themes throughout the data. Computer-Aided Qualitative Analysis Software Nvivo (produced by QSR International, Version 12.0) and Atlas.ti^®^ version 7 were used to support the management of data.

These were used to further investigate what viewers liked and disliked about the films, views about family planning discussions and couples’ counselling, and if knowledge, attitudes or intentions changed after watching the films. This article compares and contrasts the effects of viewing the second (optimised) versions of the documentary and drama films on participants’ knowledge, views, and attitudes. The development and modification of the films (based on feedback from the first round of focus group discussions) using the person-based approach will be described in a separate article and views on couples’ counselling are reported in another article [19].

### Ethical considerations

The participants were provided with explanations about the study and requested to provide written informed consent. In the case of illiterate participants, they were asked to use a thumbprint, and an independent witness signed to confirm that they gave their consent freely. Pregnant or postpartum women under the age of 18 were considered to be emancipated minors and therefore eligible to provide consent and participate in the adolescent focus groups, as per the guidelines of the Uganda National Council of Science and Technology [20]. We respected individual autonomy to participate in the study. All who consented to participate were informed about their freedom to withdraw from the study at any time without any penalties. Confidentiality was ensured by allocating non-identifiable field codes to each participant. Additionally, focus group discussion participants were asked to keep all personal information confidential.

## Results

### Characteristics of participants

Sixteen focus groups with 150 participants were carried out, in four different areas (Bwindi, Jinja, Kampala, and Mbarara). There was an average of 10 participants per focus group, with a range of six to 13 participants. Table 1 gives an overview of the demographics of the participants involved in the focus groups. In some focus groups, data for participants were not collected, leading to some gaps in the statistics. Averages were therefore made based on the information available. Participants in the focus groups on average had 2.2 children, range 0–8 (data for 24 participants missing).

**Table 1:** Demographic data of participants

	Total	M	F	Average age (years)	Educational level			
					None	Primary	Secondary	Tertiary
Women below 23	31	0	31	20.2	1	14	15	1
Women aged 23–49	40	0	40	27.8 range 23–37)	2	13	22	3
Men^a^	38	38	0	36.6 (range 21–53)	1	17	16	4
Health workers^a^	41	9	32	37.4 (range 21–56)		–	–	–

^a^Age of 24 health workers and eight men was missing

### Main themes identified

The main themes identified are the acceptability/credibility of the information provided and overall preferences for drama or documentary; changes in knowledge/beliefs about family planning; changes in attitudes towards family planning; changes in intention to discuss family planning with their partner or with others; and changes in intention to use the implant.

### Acceptability/credibility of information and overall preferences for drama or documentary

Participants felt both films provided useful information about avoiding unwanted/unplanned pregnancies. The use of local people, language and settings meant the films were more relatable than other health promotion films participants had seen, which used non-locals (and often were not in the local language), and this gave further credibility and trustworthiness. Participants said the films will help in creating awareness about family planning, they are entertaining, persuasive and attractive so people will pay attention, they are educative to both young and old people, they will attract more people to health facilities, and they can be watched as clients wait to be served by the health service provider. Health workers were keen to show the films, which can be screened repeatedly, and this would save them time in giving health education talks.

People in the documentary addressing the advantages of the implant, but also discussing the side effects they had experienced and how this had impacted them, made viewers feel that they got a more rounded view of people’s experience of the implant. They also felt that people within the documentary were giving a truthful view. Participants who preferred the documentary to drama cited that it was more educational, explained side effects and myths more clearly, and showed how it was inserted and removed.

*“I like the documentary because it exhausted everything about the implant like even the person who was addressing the film explained everything and yet in the drama, they just addressed the myth that one had said, and they never went deep to explore everything.” (22-year-old nurse, Rukiga documentary).*

*“I have liked it so much because my fellow women were explaining to us, they looked healthy, had no problems, and they showed us the implant in their arms and I am sure they haven't lied to us.” (24-year-old postnatal woman, Rukiga documentary).*

People who preferred the drama to documentary felt this was due to better communication, being persuasive, both sides of the story being shown, and being more relatable. Participants felt that the drama depicted events that happen in the community, so they could relate to the story:

*“That lady from the village, what happened to her is what takes place in the village. People from the village come with similar problems.” (Female nursing assistant, Luganda drama).*

*“I liked the drama because it shows what is going on in the community. It shows that use of the implant is good.” (19-year-old pregnant woman, Rukiga drama).*

Some participants liked both films equally; they felt that they were complementary to each other and could be used together. Some of them even suggested that the films could be merged. They also suggested that the drama should be screened first before the documentary.

*“Both films are good because both are educative. The documentary—there were things for us to learn; even the drama—we have seen how one can start using this method.” (35-year-old pregnant woman, Rukiga focus group).*

*“Between the two nurses, the nurse in the drama and her bit was just informing us about what we should do, but the documentary was real, so you should merge the two films; start with the drama and end with the documentary.” (48-year-old man, Luganda focus group).*

### Change in knowledge/beliefs about family planning

After watching the films, participants expressed views about how they caused a positive change in their knowledge and beliefs, about family planning especially concerning side-effects such as bleeding, and myths.

*“It was educative to the extent that even those who were not willing to join family planning can finally accept after being counselled by the health worker, just like the drunkard who had refused.” (22-year-old pregnant woman, Luganda drama).*

#### Side effects

Information provided was about specific side effects of the implant, how they can be managed, and how they impacted users. Addressing side-effects helped to allay these fears and led to a more positive attitude towards the implant. Participants discussed being reassured by information in the films that the side effects of bleeding and headache are likely to be temporary.

*“It has taught about what I was most worried about. I didn't know about the headache but that bleeding—they have said that it can stop, so I think I have learned and understood that. It was the only thing which was worrying me.” (38-year-old man, Luganda documentary).*

*“I have realized that bleeding after inserting the implant is not an issue because it can be managed.” (19-year-old pregnant woman, Rukiga documentary).*

*“I have learned about consulting health workers for advice on side effects management instead of listening to people’s rumours.” (30-year-old pregnant woman, Luganda documentary).*

*“I was also afraid of using it because of the side effects. I heard about it, but I have learned that they happen for a short period of time.” (29-year-old pregnant woman, Luganda drama).*

However, some participants’ concerns about side effects were not alleviated after watching the films. They wondered where the blood goes after using the implant, and one does not bleed. They were still worried about headache and loss of libido.

*“Yes, they are answering me but, that blood that is supposed to come out every month but does not by use of the implant for those years, I keep wondering where it goes and where is it kept? That is what I did not understand. I need to understand and, I keep thinking, won't it at one point come like a “river”?” (25-year-old man, Rukiga drama).*

*“Another thing, they have said that you may experience headache for 1 or 2 weeks. I think you feel bad because 2 weeks are very many while feeling headache. I am a bit worried.” (29-year-old pregnant woman, Luganda drama).*

*“They said when they get a headache and then swallow Paracetamol, they become healed, but me when I get a headache and use Paracetamol, I don't get healed.” (20-year-old pregnant woman, Rukiga drama).*

*“In the film, we have seen that implant doesn't reduce on the libido but, some of our spouses have experienced a loss of libido and increased blood pressure which they haven't shown us there. Say something about that issue.” (33-year-old man, Luganda drama).*

#### Myths and misconceptions

Participants were happy that both films were equally informative about common myths and misconceptions around family planning. Participants appreciated that after insertion of the implant, it does not move around, disappear to other body parts, or make them unable to work which had been common myths.

*“I was worried because I heard that when you have the implant, it can move in the body and disappear. When I saw the nurse removing it, I saw her removing it from exactly where she put it.” (27-year-old woman, Rukiga documentary).*

*“What I knew was that when they insert it, you do not perform tasks that require a lot of energy but, I heard those who inserted it saying that after at least 2 weeks, you can perform your duties without any problem.” (19-year-old pregnant woman, Luganda documentary).*

#### Implant insertion and removal procedure

Many potential users of implants shy away from using it since they had heard exaggerated tales of its insertion and removal. To address this concern, the documentary film included clips of the implant being inserted and removed. It was however not possible to include this in the drama. The vast majority of participants were surprised by the simplicity of the procedure, which alleviated their fears and encouraged them to use the method.

*"It was good because I thought that they make a big incision… but I have seen that it is ok." (21-year-old pregnant woman, Luganda documentary).*

*“It was practical. Some mothers think that during implant insertion, they cut the whole arm. They even think that removing it is difficult. So, when they watch this film, they will understand how it is done.” (21-year-old midwife, Rukiga documentary).*

*“What I have liked about this implant is that it was inserted in a woman while I watched, and then removed while I watched still. That means that it can be put and removed at any time, and the woman conceives. When I am in the village, I can tell them that when you use the implant, it can be removed when you want a child.” (Man, unknown age, Rukiga documentary).*

On the other hand, a small minority of participants were concerned about the pain the woman goes through during the procedure or were scared seeing the procedure, which might deter them from using the implant.

*“I did not like the part of inserting and removing of the implant because it seemed painful.” (19-year-old pregnant woman, Rukiga documentary).*

*“The way they insert the implant is disgusting. It is so catchy but irritating.” (33-year-old man, Luganda documentary).*

Although the drama did not show how the implant was inserted or removed, some participants also felt that the drama helped to address their fears and concerns about implant insertion and removal.

*“I was worried about cutting to insert it. I noticed that other women are using it so, I think that I can also use it.” (19-year-old pregnant woman, Luganda drama).*

### Changes in attitudes towards family planning after watching films

Many viewers reported a positive change in their attitudes towards family planning after watching the films. The films passed several subliminal images, including that family planning helped people to be healthy and to become wealthier. Women were particularly influenced by the idea that family planning could improve their appearance and that of their home and children.

*“It has shown us that when you give birth several times, you look unhealthy, and your home can't look good. Even if one met you, they can’t think you have those children. So when you use family planning, your home will look good, and you and your children will look good.” (28-year-old pregnant woman, Rukiga drama).*

*“The advice was good because if you don't give birth every day, you look good and healthy, and your husband also understands that you are beautiful. When you give birth frequently, your husband goes to look for the ones who are beautiful. But family planning helps you to keep young and beautiful, and your husband will keep with you.” (24-year-old postnatal woman, Rukiga drama).*

Men liked the concept of planning when and how many children to have, as they felt this would make it easier for them to support their family and organize their finances.

*“This family planning method enables one to develop himself. When you agree with your wife, you can use this method, and it works for you well—even poverty will be fought against when using this method.” (Man (unknown age), Rukiga Documentary).*

The character of the drunkard in the drama appears to have been a particularly powerful force for change, in that many men did not want to be irresponsible like him.

*“A responsible man should let his wife use the implant so that he doesn’t get in the state of failing to get resources to take care of the children because such children won't get a chance to go to school. They won't even eat well. Even the woman looks unhealthy.” (38-year-old man, Rukiga drama).*

*“What I have learned from the film is that it helps you to stop having stupid thoughts like the one the drunkard had that "the Bible says, produce and multiply.” (21-year-old man, Luganda drama).*

The drama also specifically improved attitudes towards the implant as opposed to other forms of contraception.

*“I have understood that the women who use pills are not safe. Sometimes you can go back home drunk, and maybe she hasn't gone to the health unit to pick her pills, you end up impregnating her. Yet for an implant, it can even spend 5 years which is a good method.” (Man (unknown age), Rukiga drama).*

### Change in intention to discuss family planning after watching the films

The drama was likely to encourage discussion about family planning among spouses since women need support from their spouses especially when they get side effects.

*“For me, I have seen in this film that when you do not tell your husband before using a method of family planning, it is bad. When you face challenges, he will assist you because he will be knowing the cause, and he will be patient with you.” (37-year-old woman, Rukiga drama).*

*“I have realized that discussing family planning with the husband is good. It will help keep peace in the home.” (20-year-old woman, Rukiga drama).*

Men also changed their attitudes about agreeing with their wives to use family planning after watching the drama. This is important because so many women do not have agency to choose family planning without their husbands’ consent. The question remains if men will be able to see films in health centres.

*“In the past, we as men have not been supporting family planning. Women somehow are educated, and they know something about family planning. Since the men do not understand much about it, they continue discouraging it. You find that since the woman knows about it, she goes ahead and uses it without the husband’s knowledge. Through this film, I have learned that sometimes we have to agree.” (39-year-old man, Luganda drama).*

The documentary also encouraged viewers to discuss family planning with their partner, especially the part showing a couple doing exactly this. Both men and women felt this was a convincing delivery of a message which applied to both husband and wife. The unity of the couples involved gave participants, especially women, the confidence to involve their partners and to begin using family planning.

*“Like that part where a man, a woman, and their child were seated together, I think that when you watch this film with your husband, he can allow you to use family planning method and use the implant.” (21-year-old postnatal woman, Rukiga documentary).*

*“In the documentary, we saw husband and wife seated together. The man was supporting the use of the implant.” (16-year-old pregnant girl, Rukiga documentary).*

However, a few of the viewers were sceptical about the documentary influencing them to initiate a discussion about family planning with their partners. They thought it was the role of health workers to talk about family planning.

*“If I went now and tried to give this advice to my wife, she would not like it more than if the nurse teaches her herself. Women tend to believe more in the health workers.” (Man (unknown age), Rukiga documentary).*

Participants also said that both films encouraged them to discuss family planning with others:

*“Those testifying about this implant, we have seen their faces, and we know them. They have the truth. This has made me like it, and I will even teach it to someone else in my family.” (Man, unknown age, Rukiga documentary).*

*“The film is good; I have learned something in that I can even educate like my sisters and my friends. I can also educate others.” (43-year-old man, Luganda drama).*

### Change in intention to use the implant

After watching the films, all participants were asked whether they would use the implant; all replied that they would. After seeing the documentary, women deemed it important and necessary to use the implant after delivery before being discharged home.

*"Like we are here as women, some of us are pregnant, others have just given birth. Women should reach agreement with their husbands, and they start using family planning methods immediately after birth. You can go home without it and think you will come back to the health unit after 6 weeks, and before you know it, you conceive before then, thus not spacing your children. I suggest that women should go home with methods of family planning, so that they can have healthy children." (24-year-old postnatal woman, Rukiga documentary).*

The insertion and removal of the implant particularly encouraged some participants to tell others to use the implant, since they had learned how simple the procedure was (see quotes above).

The drama also encouraged participants to use the implant, although the strongest message was the need to first discuss it as a couple.

*“I should encourage my wife to use it to have a family that we can manage.” (38-year-old man, Rukiga drama)*

*“The film was good because it has taught me that I need to first reach agreement with my husband before going for family planning to prevent chaos.” (24-year-old pregnant woman, Luganda drama).*

## Discussion

This study investigated peoples’ views about family planning films in Uganda. People generally enjoyed both films (documentary and drama) and found aspects of each interesting, educational, persuasive, and relevant to them in line with recommendations by Cadogan and others [15]. Watching the films caused a positive change in viewers’ knowledge and attitudes about the implant and their intention to discuss its use with their partner. This was mainly because films improved their knowledge about how the implant works and the procedure of inserting and removing the implants, showed the advantages of using implants and addressed their fears about side effects and myths.

After watching the films, most participants expressed a positive attitude towards family planning and intended to start using the implant. It was particularly encouraging that men also changed their attitudes, because many women do not have agency to choose family planning without their husband’s consent. This is supported by previous studies which had shown that fear of side effects and myths about family planning hinder the use of contraceptives [9, 10]. This study also answered the need for more information and education on the implants as highlighted in previous studies [10–12]. Thus, we anticipate that watching the films will lead to an increase in family planning uptake. This is in agreement with other studies showing that audio-visual media can increase the use of modern contraceptives and change family planning behaviours [10, 21–23]. Although these studies were not carried out in Uganda, they were in comparable low- to middle-income regions. However, these papers used quantitative methods and did not explore in-depth reasons for these changes. Additionally, none of these films were developed using the Person-Based Approach [13]. The production of media that is culturally appropriate and features local people in a local language means messages are more likely to have a greater impact, as shown in other areas [24]. It is hoped that these films will have a greater effect on the uptake of family planning than previous films.

Both documentaries and dramas are likely to change the knowledge, beliefs, attitudes, and practice of people towards family planning. The two styles are complementary. The drama may be more effective at changing attitudes to family planning by showing real-world situations which could not easily be included in a documentary—for example, arguments in a family and a drunkard who was initially refusing for his wife to use family planning. A documentary may be more effective at improving knowledge for example by showing real users talking about their personal experiences of side-effects, and by showing how the implant is inserted and removed. No participant was concerned about the duration of the films and in general, they were happy to see both.

### Study strengths and limitations

We conducted a large number of focus groups with a wide range of participants in different settings. During analysis, data saturation was reached in all topic areas. Additionally, the films were shown in different orders in each focus group. This minimized differences due to reduced concentration or perceptions that less information was missing in the second film if it had already been covered in the first. Another strength is the involvement of men. Their views on family planning as well as their opinion on the use of films and the message therein provide evidence that can be used to scale up interventions that involve men in family planning decisions and relevant interventions.

Despite the above strengths, the study had some limitations. In some focus group transcripts, towards the end of the discussions, it was not always entirely clear which film the participants were referring to. Also due to limited time and resource constraints, it was not possible to make some of the minor changes to the films requested by participants. The inclusion of some of the same health workers in both rounds of focus group discussions could have reduced the diversity of views; on the other hand, it was interesting to note that the same health workers were more positive about the second versions of the films than about the first versions. A further limitation was that participants were all recruited from health centres. This limited the range of views, as those who have the least amount of knowledge on family planning are not likely to visit health centres at all. However, as the intention is to show films in waiting areas of health centres, our participants are representative of the target audience. Overall a large amount of feedback and data was still generated and gave a substantial range of viewpoints.

### Implications for policy and practice

This study confirmed previous research showing that potential family planning clients are worried about side effects (both real and perceived) and there are many tales of myths and misconceptions about the use of family planning which need to be addressed. Many programs have mainly relied on radio and television programs and community health workers or Village Health Teams (VHTs) to provide health education messages, but these have never previously been developed using the person-based approach (PBA). No previous studies have compared reactions to drama or documentary style films. This study shows that both can be equally acceptable but have complementary features. A drama is good for engaging the audience, but ideally needs to be followed by a documentary to explain facts and show procedures which cannot easily be depicted in a drama.

Our films are now approved by the Uganda Ministry of Health and should be screened in waiting areas in health facilities when clients have no shortage of time and have an opportunity to ask questions afterwards when consulting health workers. They could also be shown in other venues in the community. It would be important to develop more similar films in other commonly spoken languages to reach other regions and communities in Uganda (including refugees who do not speak local languages), and also to discuss other important health issues.

### Further research

Further studies in other communities worldwide are needed to determine the degree to which our findings generalize to other communities and their healthcare systems. Although our study suggests that watching the films would increase uptake of the contraceptive implant, a cluster-randomized controlled trial would be needed to confirm this and to measure the size of the effect. Further research is underway to determine the feasibility of showing these films in health centres and other ways, for example, village health teams showing the films on a smartphone to couples at home [25]. It will be important to assess whether showing the film in health centres is sufficient to reach men (who are less likely to attend than women). Further research is also needed to determine whether the films alone are sufficient to change behaviour or whether they need to be combined with couples’ counselling by village health teams and/or health workers to reach the maximum potential effect.

## Conclusions

This study has shown that viewing both documentary and drama films (developed using the person-based approach) on family planning (with an emphasis on implants) led to a positive change in knowledge, attitudes, and intentions to use family planning. The films engaged viewers and were perceived to be educative, informative, persuasive, and entertaining. The participants’ preference was almost equally split between the drama and the documentary in the study setting. Some of the participants preferred both films. The approaches have complementary advantages and showing both may be more impactful than showing only one of the two.

## Data Availability

The full dataset generated and analysed during the current study is not publicly available to preserve the privacy of the individuals interviewed during this study. De-identified data can be made available from the corresponding author on reasonable request.

## Abbreviations

F: Female
IUDs: Intrauterine contraceptive devices
LARCs: Long-acting reversible contraceptives
M: Male
VHT: Village health team
